# Treatment outcomes from community-based drug resistant tuberculosis treatment programs: a systematic review and meta-analysis

**DOI:** 10.1186/1471-2334-14-333

**Published:** 2014-06-17

**Authors:** Pamela Weiss, Wenjia Chen, Victoria J Cook, James C Johnston

**Affiliations:** 1 School of Humanitarian Studies, Royal Roads University, 2005 Sooke Rd, Victoria, British Columbia; 2 Collaboration for Outcomes Research and Evaluation, University of British Columbia, 2405 Wesbrook Mall, Vancouver, Canada; 3 Division of Respirology, University of British Columbia, Vancouver, Canada; 4 The British Columbia Center for Disease Control, 655 West 12th Avenue, Vancouver, BC V5Z4R4, Canada

**Keywords:** Tuberculosis, Multidrug-resistant, Treatment, Community based

## Abstract

**Background:**

There is increasing evidence that community-based treatment of drug resistant tuberculosis (DRTB) is a feasible and cost-effective alternative to centralized, hospital-based care. Although several large programs have reported favourable outcomes from community-based treatment, to date there has been no systematic assessment of community-based DRTB treatment program outcomes. The objective of this study was to synthesize available evidence on treatment outcomes from community based multi-drug resistant (MDRTB) and extensively drug resistant tuberculosis (XDRTB) treatment programs.

**Methods:**

We performed a systematic review and meta-analysis of the published literature to examine treatment outcomes from community-based MDRTB and XDRTB treatment programs. Studies reporting outcomes from programs using community-based treatment strategies and reporting outcomes consistent with WHO guidelines were included for analysis. Treatment outcomes, including treatment success, default, failure, and death were pooled for analysis. Meta-regression was performed to examine for associations between treatment outcomes and program or patient factors.

**Results:**

Overall 10 studies reporting outcomes on 1288 DRTB patients were included for analysis. Of this population, 65% [95% CI 59-71%] of patients had a successful outcome, 15% [95% CI 12-19%] defaulted, 13% [95% CI 9-18%] died, and 6% [95% CI 3-11%] failed treatment for a total of 35% [95% CI 29-41%] with unsuccessful treatment outcome. Meta-regression failed to identify any factors associated with treatment success, including study year, age of participants, HIV prevalence, XDRTB prevalence, treatment regimen, directly observed therapy (DOT) location or DOT provider.

**Conclusions:**

Outcomes of community-based MDRTB and XDRTB treatment outcomes appear similar to overall treatment outcomes published in three systematic reviews on MDRTB therapy. Work is needed to delineate program characteristics associated with improved treatment outcomes.

## Background

Drug resistant tuberculosis (DRTB) is a global health concern that undermines recent successes in tuberculosis (TB) control [1]. DRTB includes both multidrug-resistant (MDR) and extensively drug-resistant (XDR) TB; MDRTB strains are resistant to the two most-effective first-line anti-TB drugs, while XDRTB is resistant to four highly effective anti-TB drugs [2]. Worldwide there are approximately 650,000 cases of MDRTB of which 10% are XDRTB [3,4]. Without significant scale-up in diagnostic and treatment capacity for DRTB, MDRTB and XDRTB could become the dominant forms of TB worldwide [1].

Treatment of MDRTB and XDRTB requires second-line anti-TB drugs that are more costly, less efficacious and more toxic than first-line drugs [4,5], and require ≥20 months of medical therapy [6]. Treatment is typically delivered using the WHO DOTS-Plus model and traditionally involves prolonged inpatient treatment that enables enhanced monitoring of adverse drug reactions, ensures adherence, and may prevent spread within the community [7,8]. Unfortunately, resource limitations often force patients to wait months for inpatient therapy, during which time they can spread to other people in their community. Inpatient therapy also increases the risk of nosocomial transmission, particularly in low-resource settings.

To address these challenges, many DRTB treatment programs have incorporated community participation in the DRTB treatment. Community-based directly observed therapy (cb-DOTS) programs are low-cost treatment programs that utilize family members, neighbours, co-workers, local health care workers (HCWs) or former patients to directly observe treatment rather than requiring hospitalizations or frequent visits to a health care facility. For drug-susceptible TB, cb-DOTS appears comparable or better than hospital-based approaches [9-11]. Many research groups have examined treatment outcomes of community-based DRTB treatment models and report good results, however to date no systematic evaluation of cb-DRTB programs has been reported in the literature. Our objective was to synthesize available evidence on treatment outcomes from community based multi-drug resistant (MDRTB) and extensively drug resistant tuberculosis (XDRTB) treatment programs.

We performed a systematic review and meta-analysis to investigate treatment outcomes in community-based MDRTB and XDRTB treatment programs. For the purpose of this study, community-based refers to treatment that occurs on an outpatient basis, and includes participation by community members in treatment delivery. Treatment outcomes were examined and pooled for analysis. Program and patient characteristics were also analyzed to determine the effect these variables had on treatment success.

## Methods

The present review have been reported according to the preferred reporting items for systematic reviews and meta-analyses (PRISMA) (Additional file 1).

### Search strategy

A methodical strategy was used to identify relevant publications. Our search strategy was modeled after Johnston et al. [12] and Orenstein et al. [13] with slight modification. The search was limited to English language publications in the EMBASE, MEDLINE, International Pharmaceutical Abstracts and BIOSIS databases and the Web of Science that were published between January 1990 and August 2012. Keyword searches were conducted on both titles and abstracts to identify relevant publications using combinations of the keywords “MDR*”, “XDR*”, “drug resistant”, “drug-resistant”, “multidrug”, “multi-drug”, “extensively”, “TB”, “tuberculosis”, “directly observed”, “DOTS”, “DOTS-Plus”, “cb-DOTS”, “treatment”, “community”, “outpatient”, “public participation”, “community-based”, “decentralized”, “home-based”, “ambulatory”, “clinic”, “community health worker”, and “CHW”. A search of EBM reviews was also conducted to determine existing systematic reviews on this topic. This included Database of Abstracts of Reviews and Effects, Cochrane Central Register of Controlled Trials and Cochrane Database of Systematic Reviews. Citations were all thoroughly reviewed and it was determined that no systematic reviews were published on this subject. Online archives of several journals were also methodically searched manually from January 1990 (when available). Journals searched included *American Journal of Respiratory and Critical Care Medicine, Clinical Infectious Disease, Chest, International Journal of Tuberculosis and Lung Disease,* and *Journal of Infectious Disease.* Bibliographic searches of identified articles were conducted to identify other relevant studies.

### Selection of studies

Relevant articles were reviewed and examined for eligibility beginning with the abstract and followed by full text review. The following inclusion criteria were applied: original study; published in English after January 1990; reported treatment outcomes on patients with culture-confirmed MDRTB or XDRTB; utilized directly observed treatment on an outpatient basis; employed community-based treatment strategies; reported treatment outcomes that would allow for comparison with other studies. Studies were excluded if they utilized only surgical interventions, reported only preliminary outcomes, routinely hospitalized patients for ≥ six months, or did not report data in a format enabling extraction.

### Methodological assessment

Two authors (P.W. and J.J.) independently assessed the methodological quality of the selected studies considered in the current review. Randomized controlled-trials, prospective cohorts, retrospective cohorts or consecutive case control studies were assessed. Publications included in this analysis reported treatment outcomes for ≥ five patients, reported results on at least 50% of patients, reported general demographic information on patients, and included community-based treatment ≥ six months in duration and total treatment duration of ≥18 months. In the case of duplicate data, the publication with the more detailed reports on treatment outcomes was included for meta-analysis. Studies were selected by one author (P.W.), with selected studies reviewed for inclusion/exclusion by two authors (P.W, J.J.).

### Treatment outcome definitions

We used treatment outcome measures defined by Laserson *et al.* and the WHO [5,14]. Patients that met the criteria for *cure* or *treatment completed* were classified as having successful treatment outcomes. Patients that met criteria for *death, treatment default*, *treatment failure* or *transfer out* were classified as having unsuccessful treatment outcomes. For data analysis, patients whose results were not available or patients that met *transfer out* criteria were placed in the *treatment default* category.

### Study characteristics

The association between treatment success and several study characteristics was examined among several subgroups. Factors examined included study year (enrollment started before 2002 versus after 2002), patient age (>14 years versus ≤14), HIV prevalence (in the cohort described) (0-2%; >2%), XDRTB infection (0%; >0%), treatment regimen (individualized; standardized), DOT location (home-based; clinic/PHC-based) and DOT provider (CHWs/HCWs only; included family/friends).

### Data analysis

Data extraction was performed by one author (P.M.) and cross-checked by a second author (J.J.). Data was analyzed using Microsoft Excel (version 14 · 1 · 0) and StatsDirect (version 2 · 7 · 9) and STATA/IC v12 · 0. Treatment outcome data (successful, default, death and failure) across all studies were pooled to measure overall treatment outcomes associated with community-based treatment. The Heterogeneity between these studies was assessed with by calculating I^2^ test. A calculated value of I^2^ > 50% indicated substantial heterogeneity. For pooling of these results, we used a more conservative random-effect model. An Egger test was used to assess for publication bias, and funnel plots were created.

To examine sources of heterogeneity, a random-effects meta-regression was performed. The dependent variable was logit-transformed DRTB treatment success (ES). All 10 studies were included in this analysis. For ES = 0 or 1, to avoid generating missing data, a small adjustment term (2n)^−1^ was applied to the logit-transformation [15]. Standard errors were adjusted in accordance. This analysis was based on a significance level at p = 0 · 05. Predictors were examined using univariate meta-regression models.

## Results

**Figure 1:**
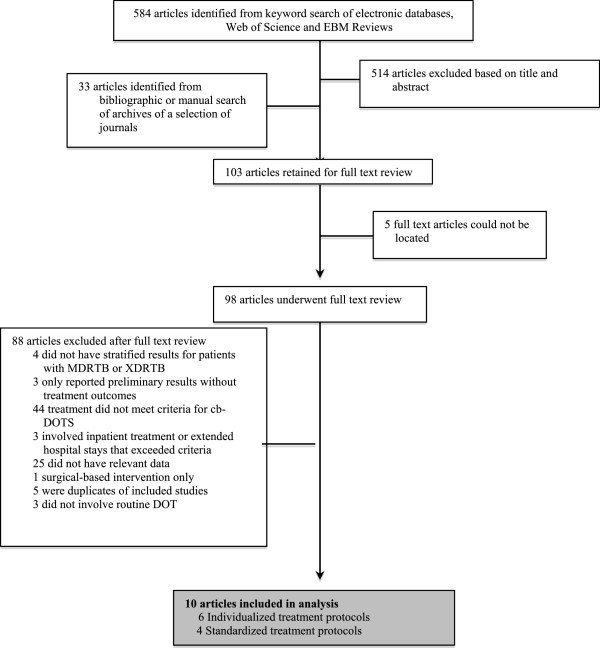
Literature search and study selection process.

Figure 1 shows the study selection process. The initial database search yielded 584 articles, while manual search and bibliographic search yielded 33 additional articles. Of these articles, 103 were retained for full text review; 88 studies were excluded for various reasons, leaving 10 articles for analysis (Figure 1) [16-25]. Overall the dates of enrollment for studies ranged from 1996–2011 and examined populations in six different countries (Table 1). Eight studies were retrospective and two were prospective.

**Table 1:** Baseline characteristics of the included studies

**Study**	**Location**	**Date**	Study type*	**Sample size**	**XDR (%)**	**HIV (%)**	**Previous therapy (%)**	**Mean resistance**
Drobac et al [19]	Peru	1999-2003	RC	27	-	-	-	-
Joseph et al [17]	India	2006-2007	PC	38	0	0	100	3 · 6
Malla et al [22]	Nepal	2005-2006	RC	175	-	-	93	3 · 7
Mitnick et al. [20]	Peru	1996-1999	RC	75	-	1 · 3	100	Median 6
Mitnick et al. [21]	Peru	1999-2002	RC	651	7 · 4	1 · 4	65	5 · 3, 8 · 4
Oyieng’o et al. [24]	Kenya	2008-2010	RC	8	0	50	100	3 · 1
Satti et al. [25]	Lesotho	2007-2011	RC	5	0	60	-	2.8
Singla et al. [16]	India	2002-2006	RC	126	-	0	100	3
Thomas et al. [18]	India	1999-2003	PC	66	1 · 5	-	100	3 · 4
Tupasi et al. [23]	Philippines	1999-2002	RC	117	-	-	96	-

*RC = retrospective cohort; PC = prospective cohort.

### Study heterogeneity

There was a high degree of heterogeneity between studies. This was not unexpected, as each study differed in terms of population characteristics and treatment model. Studies involved a mean 129 participants with a broad range of sample sizes (5–651) (Table 1). Two studies only included children under ≤15 years [19,25]. Two studies reported and included patients infected with XDRTB, with a mean of 4 · 4% (1–7). Four studies reported and included patients co-infected with HIV, with a mean of 28 · 2% (1–60). Of the eight studies that reported on previous TB therapy, 94 · 3% (65–100) of patients received previous treatment. Eight studies reported on retrospective cohorts, while two studies reported on prospective cohorts.

In terms of treatment models, six studies utilized an individualized regimen and four studies utilized a standardized regimen (Table 2). Treatment duration was expressed in different ways and varied between studies. All studies except two reported the DOTS location; these two studies described outpatient treatment. The other studies involved treatment at local health centers or decentralized clinics, local hospitals or in patient homes. The DOTS provider was reported in all studies and consisted of CHWs, HCWs, local nurses, friends, neighbours or household members. Some studies reported additional community involvement in the form of community education and support programs, the nomination of treatment support individuals and community teams that tracked patients and did home visits if any treatments were missed.

**Table 2:** Description of the treatment in the included studies

**Study**	Model*	**Treatment duration (months)**	**Drugs in regimen**^**†**^	**DOTS location**	**DOTS provider**	**Additional community involvement**
Drobac et al. [19]	I	18-24	n/a	Local health centre	CHWs, nurses	CHWs provided doses outside centre hours
Joseph et al. [17]	S	6-9, 18	6,4	Local health centre	HCWs, friends/neighbours	Health education provided for family
Malla et al. [22]	S	8-12,16-24	5,4	Decentralized clinics	Health workers	Nominated treatment support person required
Mitnick et al. [20]	I	Median 23 (0 · 4-35 · 9)	Median 6 (5-9)	Outpatient	CHWs, nurses	-
Mitnick et al. [21]	I	≥18	≥5	Health centre or patient home	CHWs	Group therapy as needed
Oyieng’o et al. [24]	S	≥6, 18	5,4	Local health centre or patient home	Local nurse, HCW, household member	Household member supervised evening dose
Satti et al. [25]	I	≥18	6	Outpatient	CHWs	Community team tracked patients and provided support
Singla et al. [16]	S	6-9, 18	5,3	Peripheral health centre or patient home	HCW, household member	Household member supervised evening dose
Thomas et al. [18]	I	≥18	5, ≥2	Village health centres, clinics or hospital	Anganwadi workers, village HCWs, private practitioners	-
Tupasi et al. [26]	I	≥6, ≥18	5, 4	Local health centre, or patient home	HCWs	Treatment partner nominated by the patient

*I = Individualized, S = Standardized.

^‡^Duration of intensive phase and continuation phase are separated by a comma. Otherwise duration represents length of treatment. ^†^Number of drugs in intensive and continuation regimens separated by a comma.

### Treatment outcomes

**Figure 2:**
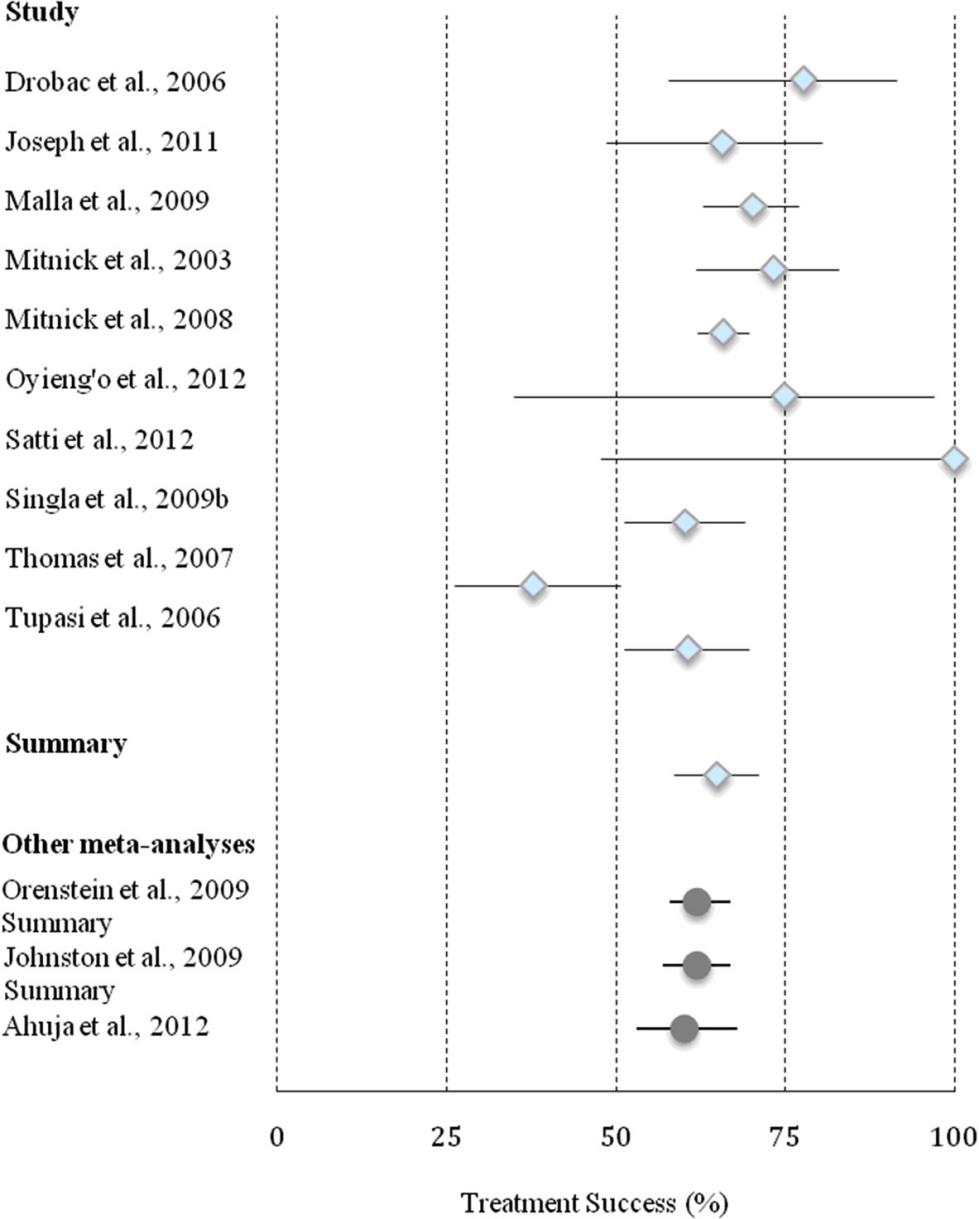
Forest plot representing treatment success with results from meta-analyses.

Overall, the 10 studies examined the treatment outcomes of 1288 DRTB patients (Table 3; Figure 2). Of this population, 65% [95% CI 59-71%] had a successful outcome (Figure 2). A total of 15% [95% CI 12-19%] of patients defaulted, 13% [95% CI 9-18%] of patients died, and 6% [95% CI 3-11%] failed treatment for a total of 35% [95% CI 29-41%] with an unsuccessful treatment outcome. Heterogeneity between studies was high (I^2^ > 50%) for all treatment outcomes except default. All pooled treatment outcome results were statistically significant (p < 0 · 05). Based on the funnel plot, there was no evidence of publication bias (Additional file 2) (Egger test p = 0 · 69).

**Table 3:** Outcomes at the end of treatment

**Authors**	**Sample size**	**Successful outcome (n)**	**Unsuccessful outcome**		
			**Default (n)**	**Death (n)**	**Failure (n)**
Drobac et al. [19]	27	21	5	1	0
Joseph et al. [17]	38	25	5	3	5
Malla et al. [22]	175	123	29	14	9
Mitnick et al. [20]	75	55	14	5	1
Mitnick et al. [21]	651	429	70	134	18
Oyieng’o et al. [24]	8	6	0	2	0
Satti et al. [25]	5	5	0	0	0
Singla et al. [16]	126	76	22	24	4
Thomas et al. [18]	66	25	16	8	17
Tupasi et al. [26]	117	71	16	18	12
Summary	129	65%	15%	13%	6%
95% CI	-	[59-71]	[12-19]	[9-18]	[3-11]
I^2^ Statistic	-	73%	49%	74%	81%
p value	-	0.0001	0.0381	<0.0001	<0.0001

### Subgroup treatment success

Treatment success among study subgroups was pooled and analyzed (Table 4). The univariate meta-regression analysis was performed to explain the source of heterogeneity. Treatment success did not differ significantly based on study year, age of participants, HIV prevalence, XDRTB prevalence, treatment regimen, DOTS location or DOT provider (Additional file 3).

**Table 4:** Treatment success among study subgroups

**Subgroups**	**Studies**	**Treatment success**
	**(n)**	**(95% CI)**
**Year study began**		
2002 or later	5	68% (59-75)
Before 2002	5	63% (52-73)
**Patient’s age**		
Included patients ≤14 years old	6	67% (54-78)
All patients >14 years old	4	65% (62-68)
**HIV prevalence**		
0-2%	4	72% (61-81)
HIV > 2%	2	85% (55-100)
Not specified or unknown	4	61% (46-75)
**XDRTB prevalence**		
0%	3	75% (55-91)
>0%	2	53% (26-78)
Not specified or unknown	5	67% (61-73)
**Treatment model**		
Individualized	6	65% (54-75)
Standardized	4	66% (60-71)
**DOTS location**		
Included home-based option	4	64% (61-68)
Clinic or public health centre only	4	63% (45-79)
Not specified	2	82% (54-98)
**DOTS provider**		
Family members, neighbours or household members sometimes utilized	3	62% (55-69)
Only CHWs, HCWs and other medical practitioners	7	66% (57-74)

## Discussion

Over the past decade, evidence has amassed from treatment programs in low-income regions to demonstrate the feasibility, safety and cost-effectiveness of cb-DRTB therapy. Our findings provide further evidence to support this once controversial model of care. Overall, treatment success was 65% [95% CI 59–71] in a population of MDRTB and XDRTB patients. The results from this study are comparable to outcomes reported in two previous meta-analyses of published MDRTB literature and one individual patient data meta-analysis (Figure 2) [12,13,26]. When compared with all treatment outcomes reported by Johnston *et al*., results were similar for treatment default (15% [95% CI 12–19] versus 13% [95% CI 9–17]), death (13% [95% CI 9–18] versus 11% [95% CI 9–13]) and treatment failure (6% [95% CI 3–11] versus 8% [95% CI 5–11]) [12].

The success of cb-DOTS programs for treatment of drug-susceptible TB has been the subject of a previous systematic review. Kangovi *et al.* evaluated 24 programs and reported an overall treatment success rate of 80 · 1% [95% CI 77.1-83.2%] [27]. Their definition for community-based therapy included DOT by a community member ‘in a location other than a health facility or TB club’. Our inclusion criteria were less rigid and included programs that delivered medication from health care facilities when associated with a form of community support.

More recently, a systematic review by Bassili *et al*. examined outcomes in ambulatory MDRTB treatment programs, comparing outcomes to those from hospital-based programs [28]. Outcomes were similar between ambulatory and hospital-based outcomes. Studies included for review did not maintain a requirement for community support. Related to inclusion/exclusion criteria, this study included only 8 studies in the ambulatory care arm, and did not include large cohorts by Mitnick, Tupasi, Singla [16,21,23]. In addition, two treatment cohorts, including the largest analyzed, were from high income countries [29,30]. Thus, findings from this review may not necessarily reflect the majority of community-based MDR-TB management.

### Study limitations

The programs analyzed in this review varied in terms of DOT delivery site and community support. DOT sites included hospitals, clinics, community health centres and patient homes. Meanwhile, community support varied, and included intense educational sessions for patients and families, working with a nominated community support person, food supplementation, and transportation support. DOTS delivery was provided by various groups, including nurses, health care workers (HCWs), community members, and family members. The variability in community delivery and community supports makes the evaluation and comparison of individual community programs difficult. However, this variability likely stems from the community-responsive design of such programs, and is likely essential for the success of cb-MDRTB programs. We attempted to identify elements of cb-MDRTB programs associated with improved outcomes, such as DOTS location or DOTS provider. Based on this analysis, however, there were no significant associations with improved treatment outcomes, possibly related to the limited sample size.

The community impact of cb-MDRTB was difficult to capture in this study. We captured individual patient outcomes associated with cb-MDRTB programs, but the effect of cb-MDRTB on treatment wait times, community and hospital MDRTB transmission, community engagement and stigma, and overall cost were not analyzed. These outcomes, however, are beginning to emerge in the MDRTB literature. For example, in South Africa, Heller *et al.* reported decreased waiting times in cb-MDRTB when compared to a traditional, hospital based program [31]. Meanwhile, Fitzpatrick and Floyd examined cost-effectiveness of four MDRTB treatment programs and found that the cost per DALY averted favours cb-MDRTB therapy [32]. Further assessments will be required to better understand the influence of cb-MDRTB programs on transmission dynamics, community perception, and other population-based aspects of TB control. In addition, the stability of cb-MDRTB treatment programs during rapid scale-up will also be an important issue given the recent expansion in MDRTB point of care diagnostic capacity [33].

We should emphasize that up to four studies from our analysis were included in previous systematic reviews, which partially accounts for their similar outcomes. These four studies, however, contribute to less than 20% of the outcomes reported in all previous analyses. We considered comparing cb-MDRTB studies to studies reporting on other types of treatment programs. Unfortunately, treatment protocols are not well-described in most studies, preventing strict classification and comparison between treatment programs. In addition, our inclusion of more recent publications may bias our results towards improved MDRTB outcomes in this cohort. Indeed, our subgroup analysis demonstrates non-significant improvement in treatment outcomes between studies starting before and after 2002. However, the five studies published in or after 2009 did not demonstrate significant differences in outcomes (data not shown). Lastly, we were limited by the number of studies available for analysis; with only ten studies and 1288 patients available for comparison, subgroup analysis was quite limited.

## Conclusion

In conclusion, this systematic review and meta-analysis of cb-DRTB therapy demonstrates that the published results of community-based MDRTB and XDRTB treatment programs appear to have adequate treatment outcomes. These results help strengthen the evidence base to support the WHO’s conditional recommendation for cb-MDRTB therapy and support recent calls for decentralized MDRTB care [34]. More research is required to examine individual and population-based effects of cb-MDRTB care: How do outcomes from home-based care compare with clinic-based ambulatory care? What community supports are essential to maintaining adherence and successful outcomes in financially strapped MDRTB treatment programs? What aspects of MDRTB diagnosis and treatment can a national TB treatment program safely decentralize? On a population level the effect of community engagement and education should be analyzed more closely, along with careful epidemiological study on MDRTB transmission. In our opinion, the call for decentralized MDRTB treatment requires a rapid but well-considered response.

## Competing interests

The authors declare that they have no competing interests.

## Authors’ contributions

PW contributed to study design, data acquisition, data analysis, manuscript preparation and editing. WC contributed to study design, data analysis, manuscript preparation and editing. VC contributed to study design, manuscript preparation and editing. JJ contributed to study design, data acquisition, data analysis, manuscript preparation and editing. All authors read and approved the final manuscript.

## Pre-publication history

The pre-publication history for this paper can be accessed here:

http://www.biomedcentral.com/1471-2334/14/333/prepub

## Supplementary Material

Additional file 1 Prisma 2009 checklist. Click here for file

Additional file 2 Funnel Plot with pseudo 95% confidence intervals. Click here for file

Additional file 3 Univariate meta-regression of DRTB treatment success. Click here for file
