# Supplementary figures and images for: Treatment outcomes from community-based drug resistant tuberculosis treatment programs: a systematic review and meta-analysis
Source: BMC Infect Dis. 2014 Jun 17;14:333. doi: 10.1186/1471-2334-14-333 (PMC4071022; doi:10.1186/1471-2334-14-333)

**Additional file 2: B:** Funnel plot with pseudo 95% confidence intervals


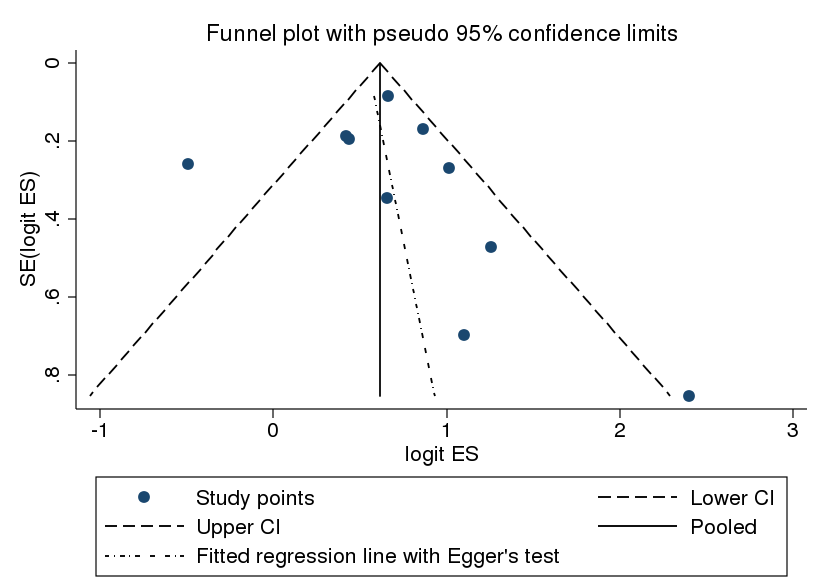

Supplement: Additional file 2 — Funnel Plot with pseudo 95% confidence intervals. [file 1471-2334-14-333-S2.doc]
