# Supplementary material for: Treatment outcomes from community-based drug resistant tuberculosis treatment programs: a systematic review and meta-analysis
Source: BMC Infect Dis. 2014 Jun 17;14:333. doi: 10.1186/1471-2334-14-333 (PMC4071022; doi:10.1186/1471-2334-14-333)
Supplement: Additional file 3 — Univariate meta-regression of DRTB treatment success. [file 1471-2334-14-333-S3.doc]

**Additional file 3: C:** Univariate meta-regression with logit-transformed DRTB treatment success

| **Subgroup** | **β** | **SE** | **Exp(B)** | **P** |
| --- | --- | --- | --- | --- |
| Study year: before vs. after 2002 | 0.31 | 0.39 | 1.36 | 0.45 |
| Age: > 14 vs. ≤14 years | -0.53 | 0.34 | 0.59 | 0.16 |
| HIV prevalence |  |  |  |  |
| Not specified | Reference | | | |
| 0-2% | 0.22 | 0.37 | 1.25 | 0.57 |
| >2% | 1.19 | 0.70 | 3.30 | 0.13 |
| XDRTB infection: |  |  |  |  |
| Not specified | Reference | | | |
| 0% | 0.35 | 0.48 | 1.43 | 0.49 |
| > 0% | -0.59 | 0.41 | 0.56 | 0.20 |
| Treatment regimen:  Individualized vs. standardized | -0.06 | 0.41 | 0.94 | 0.88 |
| DOTs location: |  |  |  |  |
| Not specified | Reference | | | |
| Included home-based | -0.77 | 0.56 | 0.35 | 0.21 |
| Clinics/PHCs only | -0.83 | 0.57 | 0.48 | 0.19 |
| DOTs provider:  included CHWs/HCWs only vs. friends/family included | 0.05 | 0.45 | 1.06 | 0.91 |
